# Case management used to optimize cancer care pathways: A systematic review

**DOI:** 10.1186/1472-6963-8-227

**Published:** 2008-11-06

**Authors:** Christian N Wulff, Marianne Thygesen, Jens Søndergaard, Peter Vedsted

**Affiliations:** 1 The Research Unit for General Practice in Aarhus, University of Aarhus, Vennelyst Boulevard 6, 8000 Aarhus C, Denmark; 2 Surgical Department P, Aarhus University Hospital, Tage-Hansens Gade 2, 8000 Aarhus C, Denmark; 3 Institute of Clinical Research, Faculty of Health Science, University of Southern Denmark, JB Winsløws Vej 12, 2 floor, 5000 Odense C, Denmark; 4 The Research Unit for General Practice, Institute for Public Health, University of Southern Denmark, JB Winsløws Vej 9, 5000 Odense C, Denmark

## Abstract

**Background:**

Reports of inadequate cancer patient care have given rise to various interventions to support cancer care pathways which, overall, seem poorly studied. Case management (CM) is one method that may support a cost-effective, high-quality patient-centred treatment and care.

The purpose of this article was to summarise intervention characteristics, outcomes of interest, results, and validity components of the published randomized controlled trials (RCTs) examining CM as a method for optimizing cancer care pathways.

**Methods:**

PubMed, Embase, Web of Science, CINAHL and The Cochrane Central Register of Controlled Trials were systematically searched for RCTs published all years up to August 2008. Identified papers were included if they passed the following standards. Inclusion criteria: 1) The intervention should meet the criteria for CM which includes multidisciplinary collaboration, care co-ordination, and it should include in-person meetings between patient and the case manager aimed at supporting, informing and educating the patient. 2) The intervention should focus on cancer patient care. 3) The intervention should aim to improve subjective or objective quality outcomes, and effects should be reported in the paper.

Exclusion criteria: Studies centred on cancer screening or palliative cancer care.

Data extraction was conducted in order to obtain a descriptive overview of intervention characteristics, outcomes of interest and findings. Elements of CONSORT guidelines and checklists were used to assess aspects of study validity.

**Results:**

The searches identified 654 unique papers, of which 25 were retrieved for scrutiny. Seven papers were finally included. Intervention characteristics, outcomes studied, findings and methodological aspects were all very diverse.

**Conclusion:**

Due to the scarcity of papers included (seven), significant heterogeneity in target group, intervention setting, outcomes measured and methodologies applied, no conclusions can be drawn about the effect of CM on cancer patient care.

It is a major challenge that CM shrouds in a "black box", which means that it is difficult to determine which aspect(s) of interventions contribute to overall effects. More trials on rigorously developed CM interventions (opening up the "black box") are needed as is the re-testing of interventions and outcomes studied in various settings.

## Background

Case management (CM) is an expanding organizational approach used to optimize the quality of treatment and care for individuals within complex patient groups [1]. Denmark has seen the launch of several CM projects intended to improve the cancer trajectory. However, a systematic literature review of the studies evaluating the effect of CM applied on cancer patient treatment and care does not exist.

Donabedian proposed the integration of aspects of structure, process and outcomes when evaluating quality of care [2], and multidimensional assessment of quality now seems to have become generally accepted [3]. Examples of inadequate cancer treatment and care categorised based on theses aspects are given below:

### Structure

Concomitantly with the expansion of treatment options (owing, among others, to advances in medical knowledge and the introduction of technological equipment) lack of specially trained staff evidently hampers better treatment[3].

### Process

Evaluations of treatment and care indicate that cancer patients and their relatives do not receive the support and information about diagnosis, treatment and postoperative course necessary for a satisfactory course and rehabilitation [4,5]. Moreover, considerable delay exists at all stages of diagnosis and treatment [6,7], which is also assumed to influence prognosis [8,9].

### Outcomes

Mortality from cancer diagnoses vary substantially between countries [10] Moreover, cancer patients and their relatives suffer from poor physical, psychological and social conditions, which could probably be improved.

Basically, the purpose of CM is to link and optimize quality and cost-effective care in both hospital and community settings [11]. CM is increasingly being regarded as a useful approach for remedying health care system inadequacies [1,11,12]. Definitions and specifications of CM-models are numerous [11,13]. The following is a mainstream definition: *" [case management] is a collaborative process that assesses, plans, implements, coordinates, monitors, and evaluates the options and services required to meet the client's health and human service needs. It is characterized by advocacy, communication, and resource management and promotes quality and cost-effective interventions and outcomes." *[14]

Approaches to describe CM models also differ. One way draws on a conception-operation framework that involves three categories: 1. a brokerage model (whose primary focus is advocacy and linking of services and needs); 2. a social entrepreneurship model (where resource and budgetary control are central), or 3. a key-worker/care coordinator model (stressing the case manager functioning within an interdisciplinary team) [11]. Other ways of describing a CM model originate in the setting (hospital-based, hospital-to-community-based or community-based) [15,16] or in the disease (disease-specific context). Some CM models rely on interdisciplinary standards of care, critical pathways or disease management programmes, whereas the case manager in other models is merely the tool mapping client care [11,17]. Finally, CM models can be specified on the basis of activities performed (basic and advanced CM) [18], the mode of contact (contact per telephone, in-person meetings, accompaniment), etc. It should be noted that a model can fall simultaneously within several different frameworks.

CM is carried out by a pro-active, supportive, facilitating, multidisciplinary health care professional (or team). The case manager, most often a nurse [11], is exclusively committed to assist patients navigate the increasingly specialized and fragmented health care system. Seamless information, communication, coordination, patient involvement and shared decision-making ensure that the patient experiences a coherent and individually tailored care pathway within the existing framework of the health care system. Delivery of the right health care resources at the right time is essential [15-17].

In spite of the absence of a proper definition of where and how to introduce CM, attention to the concept generally expands [18]. Hence, decision makers, health professionals and patient societies often support the idea of using CM. However, we need to address the question of what constitutes the scientific basis for the effectiveness of CM among patient groups suffering from cancer [19]. The purpose of this paper is to systematically identify all published randomized controlled trials of CM-like interventions applied in cancer patient care and to present characteristics, effects studied and the methodological characteristics of these interventions.

## Methods

### Literature search

We performed database searches and concurrent snowball searches with the aim of detecting all published Randomized Controlled Trials (RCTs) in which CM had been applied to people with cancer. The following databases were searched for papers published in English, Norwegian, Swedish or Danish: PubMed, Embase, Web of Science, CINAHL, and The Cochrane Central Register of Controlled Trials. Articles published all years up to August 2008 were searched for.

Prior to the search, a batch of possible keywords was recorded, Thesauruses were examined and definitions looked up. The following words were searched: "case management", "case manager", "disease management", "oncologic nursing", "oncologic nurse", "home care services", "advance practice nurse", "advance practice nursing", "advanced practice nurse", "advanced practice nursing", "advance nursing", "advanced nurse", "advanced nursing", "nursing care intervention", "nursing care interventions", "care coordination", "care coordinator", "patient navigation", "patient navigator", "system navigation", and "system navigator". The above terms were combined with "neoplasms" and "cancer".

Due to database construction differences, various combinations of MeSH, key words and text words were used in the searches. Limiting publication type to "randomized controlled trial" by the use of a check box was possible in PubMed and Embase databases, whereas the Cinahl, Web of Science and Cochrane databases were searched for RCTs by adding "..."randomly" OR "randomised" OR "randomized"" (free-text) to the search.

As an example the PubMed search is specified below:

("Case Management" [Mesh] OR "case management" OR "case manager" OR "Disease Management" [Mesh] OR "Oncologic Nursing" [Mesh] OR "oncologic nurse" OR "Home Care Services" [Mesh:noexp] OR "advance practice nurse" OR "advance practice nursing" OR "advanced practice nurse" OR "advanced practice nursing" OR "advance nursing" OR "advanced nurse" OR "advanced nursing" OR "nursing care intervention" OR "nursing care interventions" OR "care coordination" OR "care coordinator" OR "patient navigation" OR "patient navigator" OR "system navigation" OR "system navigator") AND ((("Neoplasms" [Mesh] OR "cancer") AND ((Humans [Mesh]) AND (English [lang] OR Danish [lang] OR Norwegian [lang] OR Swedish [lang])))) AND ((Randomized Controlled Trial [ptyp]))

Copies of all database searches can be obtained by contacting the author (CW).

### Study selection

We included papers on CM-like interventions that fulfilled all of the following inclusion criteria:

1) The intervention should meet the criteria for CM which includes multidisciplinary collaboration, care co-ordination, and it should include in-person meetings between patient and the case manager aimed at supporting, informing and educating the patient.

2) The intervention should focus on cancer patient care (if other diseases than cancer were included, the majority of the included patients should suffer from cancer).

3) The intervention should aim to improve subjective or objective quality outcomes, and effects should be reported in the paper.

We used the following exclusion criteria: Studies centred on cancer screening or palliative cancer care.

Data extraction was conducted (without the use of any software) in order to obtain a descriptive overview of intervention characteristics, outcomes of interest and findings. Elements from the CONSORT guidelines and checklists [20,21] were used to assess elements influencing internal and external study validity.

## Results

**Figure 1:**
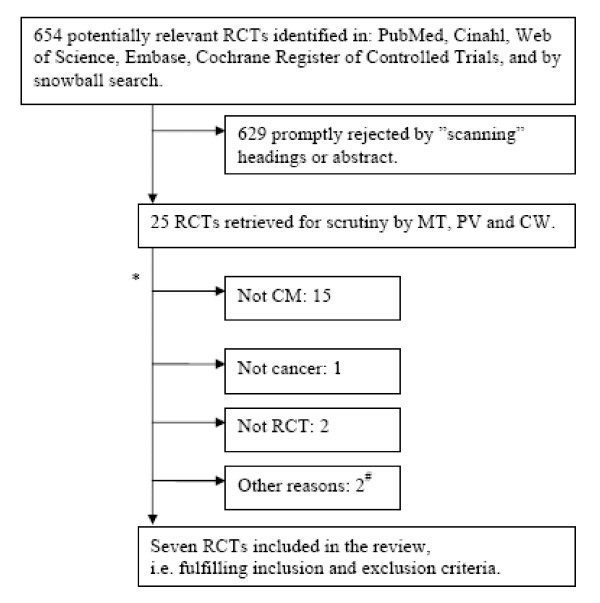
**Study inclusion and exclusion**. * Two articles were excluded for more than one reason. # Two articles reported on already included articles or components hereof.

Our search identified 654 unique papers, which after initial assessment (CW) were reduced to 25. These remaining 25 were independently scrutinised for inclusion by PV, MT and CW. When the reviewers did not agree, the paper was discussed and consensus reached. Finally, seven papers were included. Figure 1 illustrates the "flow" of papers.

The meticulous review of each included paper is reported in Tables 1, 2, and 3 (see additional file 1, 2, and 3). CW primarily filled in the Tables, and MT, PV and CW all read the included articles and checked the contents of the tables.

Characteristic elements of interventions are outlined in Table 1 (Consort items 1–5). Outcomes of interest, statistical methods, baseline data, number of participants analyzed, and effects found (Consort items 6, 8, 12, 15, 16, 17, and 18) are presented in Table 2. Table 3 presents elements of study validity found to be important: sample size, recruitment, random allocation, blinding of assessor, participant flow (Consort items 7, 14, 9, 11, and 13). Aspects of generalisability to non-participants are reported in second column points c) and d).

### Main findings

Table 1 audits the characteristics of the seven included studies. Six trials were conducted in the USA, one in the UK. Only two of the six papers termed the intervention "CM" [19,22]. The five other studies were deemed to fulfil reviewers' inclusion criteria (viz. the quoted CM definition). Interventions were classified: "advanced practice nursing" [23,24], "home care interventions" (two were tested against usual care in a three armed trial [25], "care coordination" [26], and "nurse-led follow-up" [27]. Despite the different naming, all interventions will be designated "CM" throughout the rest of this paper.

Two studies [19,23] included breast cancer patients only, two studies lung cancer patients only [25,27]. The last three included different cancer types of which one trial [26] also addressed other advanced illness (chronic obstructive pulmonary disease (COPD) and chronic heart failure (CHF)). Two studies tested hospital-to-community interventions [19,23], and three studies tested community interventions [22,24,25]. One study [27] tested a hospital (in-patient) intervention. The setting for one study was unclear, but the text implies an in-patient setting [26].

Health care professionals performed all interventions and nurses exclusively performed the intervention in five trials [19,22-24,27]. In one study, a nurse education was not a prerequisite to performing the intervention [26]. In another study [25] (the three-armed RCT), nurses were members of the intervention teams which included various professions.

Six studies described some sort of model, manual and/or use of supportive tools, e.g. assessment tool or checklist (Table 2). The sixth study [25] reported no such use of manual or tools, and offered no precise description of intended and performed intervention.

The patients' GPs were only expressly mentioned in the study from the UK [27]. This study also evaluated the intervention in terms of the GPs' satisfaction.

Table 2 presents outcomes studies. First of all, it is noteworthy that only three articles [19,24,27] mentioned which outcomes were primary and which were secondary. Since there is no overlap of outcomes studied (tool and methods of assessment) between the trials, no synthesis of effects can be made.

To further evaluate the effects, outcomes were divided (our own categories) into objective and subjective (= patient reported) elements. Objective elements studied included: survival [24,27], received therapy [19], physical function (arm function) [19], advance directives and "do-not-resuscitate and intubate"-wishes [26], service use and hospitalization [22,25,27], and costs [23,26,27]. Subjective elements studied included: patient-reported needs and symptoms [22,25], patient-reported "quality of life" [22,24,27], patient satisfaction and evaluation of the decision-making process [19,26,27], and relative-reported problems experienced in the interaction with the health care system [26].

When categorising outcomes according to the above criteria and taking nothing else into account, some dimensions of Quality of Life appeared to be improved. Similarly, all three papers reporting patient satisfaction reported improvements.

Looking at Table 3, it is obvious that validity elements of interventions were inadequately reported in most papers. Sample size measures were only reported in one paper [27], and one or more of the following elements were only poorly described: recruitment, allocation concealment method and blinding of assessor. Moreover, patient flows were incomprehensible in all papers except for one [27], which also included a flow diagram. All articles analysed whether randomization was successful regarding baseline covariates (Table 2).

## Discussion

### Principal findings

Only seven studies evaluating the effect of CM or a CM-like-intervention applied to cancer care were found.

Studies diverged much in terms of target group, intervention setting, outcomes measured and findings.

Some of the included studies reported improvements in patient satisfaction and dimensions of quality of life measures. However, the methods used to measure the effects were all different (tool and method of assessment) and validity of many of the tools was poor or unknown.

All in all, overall study validity was inadequate. Due to the heterogeneity of the studies found, it was hardly possible to score validity in a simple measure or to make a meta-analysis of the results.

Thus, no conclusions can be made about the effect of CM on cancer patient care on the present basis.

### Strengths and weaknesses of this review

The weak definition of CM and detection of interventions not specifically labelled "CM" but imitating CM forced us to search for more terms than just "case management" and "case manager". Papers on patient and system navigation were considered to conceal CM interventions and were accordingly included. However, no navigation papers passed the study selection criteria. Our search made us realize that navigation studies primarily address screening and follow-up of abnormal screening findings among poor, vulnerable people [28].

Our widened search strategy is regarded as a strength, but it also blurred the convenient clear-cut boundary between which interventions to include and which to exclude. Despite the fact that the intervention by Mor et al [22] was one of the only two included articles categorised "CM", the tested educational short-term CM intervention included very few multidisciplinary components which normally makes it possible to distinguish CM from other nursing care.

Palliative care where the focus is on "care-alleviating symptoms and not on curing the underlying disease" is regarded as an exclusive, medical and research discipline (with supportive elements resembling CM) for which reason such studies were not included [29]. Also, the rationale for not including palliative care CM intervention was rooted in our perception that a patient in a care situation involving diagnosing, treatment and follow-up and facing potential cure is experiencing problems other than those faced by a palliative care patient. Thus, our aim was to illuminate CM interventions targeting cancer patients early in their disease trajectories. It should be emphasized that our decision is not tantamount to claiming that it is irrelevant to test CM-like interventions in the palliative care setting.

Publication bias and inclusion of RCTs only may have reduced the number of papers found.

Publication bias is always a problem when conducting systematic reviews and performing meta-analyses. Theoretically, the temptation not to publish negative results of a study may be greater in the case of complex interventions than simple interventions because it is more difficult to describe the components of a complex intervention.

The present review is delimited to RCTs because this research design is considered to be superior to other trials when it comes to trusting reported effect estimates [30,31]. On the other hand, a decision to include RCTs only may be questioned when the aim is to evaluate complex intervention of which CM form part because of possible reproducibility problems regarding intervention content. It may be difficult to reproduce outcomes when the intervention tested is poorly described. MRC has set up a framework for developing complex interventions to be tested in the RCT [32,33]. When MRC's framework is followed, the RCT design can be regarded as a "gold standard", also for complex interventions.

None of included papers mentioned MRC's framework, but several made an effort to minimize the "black box" [18,34].

Manuals, tools and accounts of actually conducted activities (type and dosage) are essential to minimize the "black box". As mentioned, six studies described some sort of model, manual and/or use of supportive tools, e.g. assessment tool or checklist (Table 2). Moreover, three papers described attempts to check which CM activities had actually been conducted. Engelhardt et al [26] described that care coordinators completed checklists which afterwards were checked for intervention integrity. McCorkle et al [24] calculated and grouped nursing intervention (assessment, education, etc.) and the work by Goodwin et al [19] was succeeded by a paper based on intervention contacts [35].

### Comparisons with other studies

Summaries of nursing and CM interventions have previously been made, but this is the first review of CM targeted at cancer patients. A literature review from 2004 on evidence-based nursing interventions applied to older adults who had cancer [36] concluded that the body of literature was small and heterogeneous. Among the studies summarized, two [19,24] were also included in our review.

Other reviews of CM seem to originate in the disease or the setting.

Two reviews of disease-specific CM have been conducted: A review of CM for people with diabetes concluded that CM was effective in improving both glycaemic control and provider monitoration of glycaemic control for adults with type 2 diabetes both when delivered in conjunction with disease management and when delivered with one or more additional educational, reminder, or support interventions (setting: U.S. managed care) [1].

A review of nurse-led disease management for people with chronic obstructive pulmonary disease (COPD) concluded that: "There is little evidence to date to support the widespread implementation of nurse led management interventions for COPD, but the data are too sparse to exclude any clinically relevant benefit or harm arising from such interventions" [37].

Two papers on in-patient/hospital (setting-specific) CM were found: A meta-analysis from 2005 on the effect of CM on hospital length-of-stay (LOS) and readmission concluded that hospital-based CM was not effective in reducing LOS and the percentage of patients readmitted at least once. The meta-analysis included 12 experimental studies, none of which focused on cancer patients [38]. Similarly, a research synthesis of in-patient CM (from 1998) did not support that effectiveness was improved in terms of patient and provider satisfaction, quality-of-care, cost savings and length of stay (the study included 13 quasi-experimental and five randomized trials) [39].

## Conclusion

The purpose of CM is to optimize individual cost-effective quality care for patients suffering from chronic and/or complex diseases.

Generally, reviews on the effects of CM in somatic care have found inconclusive positive effects.

This is the first review studying CM applied in cancer patient care. Due to the scarcity of papers included (seven), significant heterogeneity of CM interventions and effects studied, and the methodological inadequacies, no conclusions on the effects of CM in cancer patient care can be made.

Further evaluations of CM in cancer patient care are needed. Future research needs to focus on the elimination of the "black box" through thorough descriptions and reporting of interventions. Instead of starting from scratch, CM developers are recommended to build research on existing models, outcome-tools and complementary research findings. Additionally, re-testing interventions in various settings could be interesting. Following MRC's framework [32] when designing and evaluating complex interventions is recommended.

## Competing interests

The authors declare that they have no competing interests.

## Authors' contributions

CW and PV initiated and conceptualised the study. CW, MT and PV developed the method. CW and MT received help from librarians and together searched the databases. Studies were selected by CW, MT and PV. CW primarily filled in the Tables, but MT, PV and CW all read the included articles and checked the contents of the tables. CW, PV, MT and JS prepared the manuscript.

## Funding

CW and MT are financed by The Novo Nordisk Foundation grant "Coherent treatment for cancer patients".

## Pre-publication history

The pre-publication history for this paper can be accessed here:



## Supplementary Material

Additional file 1 **Table 1**: Characteristics of the case management models in the seven included papers. Click here for file

Additional file 2 **Table 2**: Randomization, data collection, analyses and results. Click here for file

Additional file 3 **Table 3**: Important methodological aspects adapted from CONSORT [20,21]. Click here for file
