# Supplementary material for: Case management used to optimize cancer care pathways: A systematic review
Source: BMC Health Serv Res. 2008 Nov 6;8:227. doi: 10.1186/1472-6963-8-227 (PMC2596122; doi:10.1186/1472-6963-8-227)
Supplement: Additional file 1 — Table 1: Characteristics of the case management models in the seven included papers. [file 1472-6963-8-227-S1.pdf]

Table 1: *Characteristics of the case management models in the seven included papers*

Ref.	Authors, publication year, country, setting and location	a) Aim<br>b) Patients and cancer type<br>c) Name of intervention<br>d) CM setting<br>e) Control group exposure	a) Contact mode<br>b) Quantity of intervention<br>c) Intervention duration	a) Numbers of case managers<br>b) Cm education<br>c) Cm training in the CM-model<br>d) CM manual, tools, or the like	Effects studied
[19]	Goodwin, 2003, USA, 13 community and two public hospitals in southeastern Texas (multicenter trial)	a) To assess the efficacy of nurse case management (NCM) in improving the medical care given to community-living older women diagnosed with breast cancer.<br>b) Women aged 65 and older with newly diagnosed breast cancer.<br>c) Nurse CM<br>d) Hospital-to-community.<br>e) ? (usual care?)	a) Home visits, telephone conversations, assist the patient at physician appointments, visiting the patient at hospital, and contacts made at other community locations.<br>b) Patient needs determined the frequency of contact, minimum contact during the intervention period was at least one in-person assessment and monthly telephone calls.<br>c) 12 months from first contact.	a) Three case managers<br>b) Baccalaureate degree registered nurse with previous experience with CM in other settings.<br>c) 40 hours of training and education in treatment, complications, community resources, assessment, communicating methods, etc.<br>d) A checklist and several assessment tools (not used or analyzed by investigators)	Primary: cancer-specific therapies received<br><br>Secondary: patient evaluations of the decision-making process; arm function on affected side.
[23]	Ritz, LJ et al, 2000, USA,<br>One hospital in an integrated health care system in a mid-western suburban community.	a) To evaluate the quality of life (QoL) and cost outcomes of CM on women with newly diagnosed breast cancer.<br>b) Women, 21 years of age and older, newly diagnosed from breast cancer.<br>c) Advanced practice nursing<br>d) Hospital-to-community<br>e) "standard medical care"	a) During clinic visits, hospital, by telephone, and home visits.<br>b) Patient, family and CM need-determined. CM on-call all days during the daytime.<br>c) ?	a) Two advanced practice nurses<br>b) Registered nurse with a master's degree in nursing who has in-depth knowledge and skill in the care of the patient population.<br>c) ?<br>d) Manual not mentioned, but model developed on Brooten cost-quality model (ref), but modified and ONS Standards of Advanced Practice (ref).	Quality of Life measures<br>Cost data
[25]	McCorkle, R. et al, 1989, USA, subject recruitment from 19 hospitals and one	a) To test the effects of two different home care treatment regimens against usual care on the psychosocial well-being of patients	a) Home visits (weak description)<br>b) ?<br>c) 24 weeks.	*OHC:*<br>a) ? b) Nurses with master degrees c) ? ("trained to give personalized care to persons with	Patient Psychosocial Responses<br>Number of hospitalizations<br>Length of Stay (LOS)

Ref.	Authors, publication year, country, setting and location	a) Aim b) Patients and cancer type c) Name of intervention d) CM setting e) Control group exposure	a) Contact mode b) Quantity of intervention c) Intervention duration	a) Numbers of case managers b) Cm education c) Cm training in the CM-model d) CM manual, tools, or the like	Effects studied
	radiation outpatient facility; King County, Washington	with lung cancer.<br>b) Homebound patients suffering from lung cancer, stage II or higher.<br>c) home care interventions: Specialized oncology home care program, and Standard home care program<br>d) Community<br>e) “traditional treatment by patient’s physicians”		advanced cancer and their families “) d) ?<br>*SHC:*<br>a) ? (a team) b) An interdisciplinary team of health professionals including registered nurses; c) ? d) ?	
[24]	McCorkle, R et al, 2000, USA, out-patient setting at a Comprehensive Cancer Center in south-eastern Pennsylvania	a) To analyse whether follow-up by an advanced practice nurse can improve survival when compared to patients in an ambulatory setting.<br>b) Patients aged 60 years or older newly diagnosed with and operated from a solid tumour (different types) having an anticipated survival of 6 months or more (primary surgical removal of cancer only).<br>c) Advanced practice nurse specialized home care intervention.<br>d) Community<br>e) “usual follow-up care in an ambulatory setting”	a) Home visits and telephone.<br>b) Pre-determined home visits (three) and telephone calls (five) + according to patients’ needs. APNs were available on a 24-hours basis.<br>c) 4-weeks immediately after surgery and hospitalization	a) ?<br>b) Advanced practice nurses (are masters prepared clinicians in oncology).<br>c) ?<br>d) A standardized protocol consisting of standard assessment and management guidelines, doses of instructional content and schedules of contacts.	Primary: Length of survival<br><br>Secondary: Identify psychosocial and clinical predictors of survival
[26]	Engelhardt, JB et al, 2006, USA, three Dep. of Veterans Affairs Medical Centers (=VAMCs), a home care org., and two Managed Care Org.	a) To evaluate the Advanced Illness Coordinated Care Program (AICCP) on patient and surrogate satisfaction with health care and provider communication, Advance directive (=AD) wishes and health care costs.<br>b) Patients suffering from advanced illness (Specified cancer diagnoses	a) In-patient meetings (?)<br>b) 6-session format, but individualized. Patients could schedule extra meetings.<br>c) ?	a) ? (6 sites)<br>b) Nurses, nurse practitioners, or social workers familiar with institutional policies and who had ongoing relationships with providers (existing personnel who were replaced from normal duties).	Patients’ evaluations of patient/provider communication, satisfaction with care, and attitudes about participation in treatment planning.<br>Surrogates´ experiences with the health care system.<br>Costs

Ref.	Authors, publication year,<br>country, setting and location	a) Aim<br>b) Patients and cancer type<br>c) Name of intervention<br>d) CM setting<br>e) Control group exposure	a) Contact mode<br>b) Quantity of intervention<br>c) Intervention duration	a) Numbers of case managers<br>b) Cm education<br>c) Cm training in the CM-model<br>d) CM manual, tools, or the like	Effects studied
		and advanced COPD and CHF patients)<br>c) The Advanced Illness Coordinated Care Program (care coordination)<br>d) ? (in-patient only?)<br>e) "usual care"		c) Training and reviewed assigned readings, including the AICCP training manual.<br>d) Manual, checklists, and worksheets.	Advance directives (AD) and "do-not-resuscitate and intubate (DNR[I])" wishes.
[22]	Mor, V et al, 1995, USA,<br>two hospital based chemotherapy clinics and eight private medical oncology practices	a) To evaluate effect of a short-term, educationally-oriented CM model for chemotherapy patients.<br>b) Residents 21 years of age or more initiating a new course of chemotherapy (different cancer types).<br>c) Nurse CM<br>d) Community<br>e) Not mentioned (usual treatment?)	a) Home visits and telephone calls.<br>b) Pre-determined initial and termination home visit (about 10 weeks later), and telephone calls at 2-week intervals. Patient could contact Cm for assistance for up to 3 months..<br>c) 3 month follow-up period.	a) ?<br>b) Nurse<br>c) ?<br>d) Resource database (information about community service agencies, cancer-specific disease and treatment information).	Unmet needs<br>Symptom severity<br>Several dimensions of QoL<br>Formal service utilization
[27]	Moore, S et al, 2002, UK, one specialist cancer hospital and three local cancer units	a) To assess the effectiveness of a nurse-led follow up in the management of patients with lung cancer.<br>b) Lung cancer patients who had completed their initial anticancer treatment and were expected to survive for at least three months.<br>c) Nurse-led follow up<br>d) Hospital CM<br>e) "conventional medical follow-up"	a) Over telephone or in a nurse-led clinic.<br>b) Telephone assessment or clinic appointment two weeks after baseline, then every four weeks while patient is stable. Open access through clinic, telephone, and message pager service.<br>c) ?	a) Two<br>b) Clinical nurse specialist<br>c) Observing outpatient lung cancer clinics and shadowing medical consultants. Regular clinical supervision sessions were given.<br>d) Reference to published article describing the model.	Primary: QoL and patients' satisfaction<br><br>Secondary:<br>Overall, Symptom-free, and Progression-free survival.<br>GPs' satisfaction<br>Service use

?: Not to be found in the article
Cm: Case manager, CM: Case management
OHC: Specialized oncology home care program, SHC: Standard home care program, OC: Usual office care
AICCP: Advanced Illness Coordinated Care Program, APN: Advanced practice nursing
COPD: Chronic obstructive pulmonary disease, CHF: Chronic heart failure
