# Supplementary material for: Case management used to optimize cancer care pathways: A systematic review
Source: BMC Health Serv Res. 2008 Nov 6;8:227. doi: 10.1186/1472-6963-8-227 (PMC2596122; doi:10.1186/1472-6963-8-227)
Supplement: Additional file 2 — Table 2: Randomization, data collection, analyses and results. [file 1472-6963-8-227-S2.pdf]

Table 2: *Randomization, data collection, analyses and results*

Ref	a) Randomization type<br>b) N, n-intervention, and n-control	Outcomes		a) Statistical methods<br>b) Randomization evaluated? (yes, no)<br>If yes: variables, potential differences and possible adjustment performed noted?<br>c) Numbers included in analyses<br>d) Intention-to-treat analyses (yes/no/not mentioned)	Main results
		**Outcomes of interest*<br>(Time of measure)**	**Outcome source<br>(Validity account#)**		
[19]	a) Stratified, cluster (stratification at surgeon level (experience of surgeon's breast cancer practice); Within each stratum randomization was performed in blocks of four.<br>b) N(Surgeons)=60; N(Patients)=335, n-CM=169, n-control=166	Primary: cancer-specific therapies received<br>(after 6 months)<br><br>Secondary: patient evaluations of the decision-making process; arm function on affected side.<br>(2 (and 12) months after diagnosis.)	Primary: medical records audit (A summary measure of receipt of appropriate therapy was created based on published consensus recommendations; ref)<br><br>Secondary: home interview based on pilot tested questionnaires on logistics, decision-making, satisfaction and tamoxifen prescription (?) and objective assessment of arm functions (?).	a) Differences in baseline characteristics and in outcomes between control and intervention groups were assessed using chi-square. (Cluster effect at surgeon level was adjusted for.)<br>b) Yes. No difference found (demographics, cognitive function, and stage of disease)<br>c) Primary outcome: n-CM: 169 and n-control 166<br>Secondary outcomes: ?<br>d) Yes	Primary: More women in the intervention group saw a radiation oncologist at their initial evaluation (36.0 vs. 19.3%, P=0.006), received breast-conserving surgery (28.6 vs. 18.7%, p=0.031) and radiation therapy (36.0% vs. 19.0%; P=0.003).<br>Secondary:<br>Intervention group was significantly more satisfied (more components; p<0.05) and had significantly more normal or near-normal range of arm motion (93 vs. 84%, p=0.037).<br>(Several subgroup analyses: "Women with poor social support were most likely to benefit from the nurse CM intervention.")
[23]	a) Simple, two-arm randomization.<br>b) N=210, n-int=106, n-control=104	-Quality of Life (QoL)<br>(At enrolment + 1, 3, 6, 12, 18, 24 months after enrolment.)<br><br>-Cost data<br>(24 months after date of diagnosis).	-QoL measured with three self-administered questionnaires:<br>1. MUIS: uncertainty<br>2. POMS: mood<br>3. FACT-E: well-being/ QoL on six dimensions.<br>(yes, all validated + ref)<br>-Charges and reimbursements were collected from billing systems.<br>Length of hospitalization and number of visit to health care provider were recorded.	a) -Univariate analyses of QoL data: t-test + chi-square /Fisher's exact test. Multiple regression for repeated QoL measures using baseline scores as a covariate.<br>-Costs: Univariate analysis + multivariate regression.<br>b) Yes (variables: demographics and disease characteristics). Difference found: Intervention group women had lower histology (p=0,04) and more received adjuvant hormone therapy (p=0,03); adj. performed.	Uncertainty: Intervention group had less uncertainty at 1, 3 and 6 months (p<0.05). Effect size not specified.<br>Mood and well-being: no sign. diff. between int. and control group.<br>Overall costs: no difference found including subgroup analyses.<br>(Some subgroups benefitted significantly from APN, e.g. unmarried women and women with no family history of breast cancer).

Ref	a) Randomization type<br>b) N, n-intervention, and n-control	Outcomes		a) Statistical methods<br>b) Randomization evaluated? (yes, no)<br>If yes: variables, potential differences and possible adjustment performed noted?<br>c) Numbers included in analyses<br>d) Intention-to-treat analyses (yes/no/not mentioned)	Main results
		Outcomes of interest* (Time of measure)	Outcome source (Validity account[#])		
			Cost of APN services were based on time logs.	c) QoL: ?<br>Cost data: N=152 (n-int=78, n-contro=74; 58 excluded because of missing data)<br>d) Not mentioned	
[25]	a) Three-arm simple randomization<br>b) N=166, Numbers assigned to each of the "arms" N/A.	-Measures of Patient Psychosocial Responses (Five interviews at 6-week interval; first before group assignment.)<br><br>-Number of hospitalizations<br>-Length of Stay (LOS) (continuously through 24 weeks).	-Psychosocial responses: Interview questionnaire (in-person or telephone ?); Scales: Symptom distress (The Symptom Distress Scale); Pain(McGill-Melzack Pain Questionnaire); Current Concerns (Weisman and Worden's Inventory of Current Concerns); Mood state (Profile of Mood States)<br>Functional status (General Health Rating Index) (ref to all above)<br><br>-A Medical Record Review Instrument was developed.	a) Primary analyses: repeated measures and analysis of variance for each dependent variable (univariate mixed model and multivariate model). Plot of means for the core measures.<br>b) Yes. No difference on demographics, Starting points for depending variables were discrepant for which reason adjustment was performed (Potential bias of adj. was discussed).<br>c) Patient psychosocial responses: 78 patients completing four interviews (numbers in each group not stated).<br>Number of hospitalizations: 77 of 78 completing four interviews (n-OHC=24, n-SHC=27, n-OC=26).<br>LOS: 52 (had been hospitalized) of 78 completing four interviews (n-OHC=14, n-SHC=18, n-OC=20).<br>d) Not mentioned	Psychosocial Responses : Significant difference between the profiles of the two nursing groups and the office care group with regards to adjusted Symptom Distress (P=0.03) and adjusted Enforced Social Dependency (P=0.02) in favour of home care nursing.<br>The OC group rather steadily reported improved health perceptions over time, whereas the two treatment groups reported worse health perceptions ($p<0.05$).<br>No of hospitalizations and LOS: No significant differences

Ref	a) Randomization type<br>b) N, n-intervention, and n-control	Outcomes: Outcomes of interest* (Time of measure)	Outcomes: Outcome source (Validity account#)	a) Statistical methods<br>b) Randomization evaluated? (yes, no)<br>If yes: variables, potential differences and possible adjustment performed noted?<br>c) Numbers included in analyses<br>d) Intention-to-treat analyses (yes/no/not mentioned)	Main results
[24]	a) Simple randomization.<br>b) N=375, n-intervention=190, n-control=185	Primary: Length of survival (up to 44 months of follow up)<br>Secondary: To identify psychosocial and clinical predictors of patient survival (i.e. depressive symptoms, symptom distress, functional status, co-morbidities, length of hospital stay, age, and cancer stage).<br>(baseline, 3, and 6 months)	Survival status was ascertained by letter, telephone, or death certificates (?)<br>Demographics: “obtained at accrual” (?)<br>Stage of disease: Surgical pathology reports and physician’s discharge summary (?)<br><br>Psychosocial questionnaires : Center for Epidemiological Studies-Depression Scale (CES-D), Symptom Distress Scale (SDS), and Enforced Social Dependency Scale (ESDS) (ref to all)	a) Stratified log-rank test was used to compare groups. Kaplan-Meier curves stratified by stage of disease at diagnosis.<br>Cox’s proportional hazards regression model to compute adjusted hazard ratios (=HR; Proportional hazards assumption was Schoenfeld tested)<br>b) Yes (demographics and clinical variables; more late stage patients in intervention group (p=0,013). Adjusted and stratified analyses performed.<br>c) Survival status for all 375 included patients were obtained<br>Psychosocial questionnaire responderse: time 0: n-int=190 n-UC=185; time 3 months: n-int=163, n-UC= 153; time 6 months: n-int=158, n-UC=147).<br>d) Not mentioned	Non-stratified analyses revealed no difference in survival status between groups (p=0,129).<br>Stratified analyses:<br>Late-stage patients’ 2-year survival were 66.7% in int. group vs. 39.6% in control group (p<0.05).<br>Adjusted for psychosocial and clinical covariates:<br>Usual care had death-HR=2.04 (95% CI 1.33-3.12, p=0.001)<br>Late stage usual care patients had adjusted death-HR=4.55 (CI 2.92-7.08; p<0.001)<br><br>Outcomes of psychosocial questionnaires were not mentioned at all in results paragraph.

Ref	a) Randomization type<br>b) N, n-intervention, and n-control	Outcomes		a) Statistical methods<br>b) Randomization evaluated? (yes, no)<br>If yes: variables, potential differences and possible adjustment performed noted?<br>c) Numbers included in analyses<br>d) Intention-to-treat analyses (yes/no/not mentioned)	Main results
		**Outcomes of interest*<br>(Time of measure)**	**Outcome source<br>(Validity account[#])**		
[26]	a) Block-randomization (blocks of 10; rationale not outlined)<br>b) N=275; n-AICCP=133, n-UC=142 ; N-surrogates (relatives)=168, n-sAICCP =76, n-sUC=92.	-Patients' evaluations of patient/provider communication, satisfaction with care and attitudes about participation in treatment planning (enrolment, at 3 and 6 months)<br><br>-Surrogates´ experiences with the health care system. (3 months post-enrolment.)<br><br>-Costs (end of study)<br><br>-Advance directives (AD) and do-not-resuscitate and intubate (DNR[I]) (enrolment, 3 and 6 months)	Patient/provider communication, satisfaction with care: Investigator-constructed, 10-item scale (?, but "reliability tested on enrolment")<br>Participation in treatment planning was assessed by a single item (?; "asked" – questionnaire or interview?)<br><br>Surrogates´ experiences (problems in 7 domains were averaged to create a single overall rating): Modified EOL Family Interview (ref; "asked" - questionnaire or interview?)<br><br>Costs: Program contact, salary, and overhead costs collected from 3 sites (the VAMC patients). Other costs: medical records for VAMCs patients.<br><br>AD and DNR(I): VAMC participants' medical records	a) -Patients' evaluations: Scores were examined for effects of group, time, and group-by-time interaction using a random effects regression model.<br>-Surrogates' exp.: Post-intervention scores t-test compared.<br>-AD: Chi-square comparison and t-test. Kaplan-Meier curves comparison of group membership and time to completion of ADs.<br>-Costs: F test<br>Effect sizes were calculated for most outcomes.<br>b) Yes, (Patients' demographics and diagnoses (and later survival), no diff.; surrogates: No of participants, sex and relationship: no diff.)<br>c) Patients and surrogates evaluations: ?<br>Mean per case AICCP costs: Data for 70 VAMC patients. Other costs:169 VAMC patients (AICCP=93, UC=76).<br>AD etc: data on 180 VAMC patients (AICCP= 85 and UC=95)<br>d) Yes, all outcomes (18 patients crossed over to AICCP, two patients crossed over to UC. Some VAMCs inpatient units implemented AICCP as usual care during the study)	Patient satisfaction with care: Significant group-by-time interaction in favour of the AICCP group (Effect size 0.18, *P* = 0.03).<br>(Effect size is the ratio of the estimated treatment effect.)<br>Surrogates post-test scores: Fewer problems (with the spiritual and emotional support delivered) reported by AICCP surrogates than UC surrogates (effect size 0.39, p=0.03)<br>Costs: no stat. sign diff.<br>AD: Median time to completion of first AD: AICCP=46 days vs. UC= 238 days (log-rank P=0.02)<br>Proportion of patients having completed at least one AD, and the mean numbers of ADs per patient were sign. higher for the AICCP group at both 3 and 6 months (p=0.01).

Ref	a) Randomization type<br>b) N, n-intervention, and n-control	Outcomes		a) Statistical methods<br>b) Randomization evaluated? (yes, no)<br>If yes: variables, potential differences and possible adjustment performed noted?<br>c) Numbers included in analyses<br>d) Intention-to-treat analyses (yes/no/not mentioned)	Main results
		**Outcomes of interest*<br>(Time of measure)**	**Outcome source<br>(Validity account[#])**		
[22]	a) Stratified randomisation (six strata; three strata based on unmet need status, and two strata based on gender).<br>b) N=259, n-CM=130, n-control=129	-Unmet needs (assessed by patients)<br>-Reported symptom severity<br>-Several dimensions of QoL<br>-Formal service utilization<br>(Data collection: At baseline, at 3 and 6 months)	-Aspects of daily living (three unmet needs-categories) (ref);<br>-Standard questions on symptom severity (?)<br>-Spitzer's physical "QoL Index"(ref), five-item mood state score from SF-36 (ref), and a specially developed 4-item scale measuring patient experienced disruptions in treatment (?)<br>All above: Telephone interviews<br>-Service utilization: Patients' reports and audit of patients' medical records.	a) Chi-square and analysis of variance to test differences between intervention and control groups<br>b) yes (no difference found on baseline demographic, medical and need status)<br>d) 3 months: n-CM=109, n-control=108, 6 months: n-CM=93, n-control=92<br>e) Not mentioned; unclear if 11 CM group patients who refused CM services were followed up and in which group they were analysed (?)	No statistically significant differences were observed on any outcome measure for the overall sample as well as for selected "at-risk" patient subgroups.
[27]	a) Stratified randomisation according to hospital and treatment intent (rationale and numbers of strata not outlined)<br>b) N=203 (n-nurse led follow-up=100, n-control=103)	Primary: QoL and patients' satisfaction at three months (assessed at baseline, 3, 6, and 12 months)<br><br>Secondary:<br>Overall survival, Symptom-free survival, Progression-free survival.<br>GPs' satisfaction (at the end of study participation).<br>Service use (3, 6 and 12 months) and cost effectiveness	-EORTC QLQ-C30 and module about lung cancer. (ref)<br>-Patient satisfaction questionnaire incorporating three validated measures and tested in a pilot study (ref)<br><br>No information on source of secondary outcomes.	a) QoL + satisfaction: Mann-Whitney U test<br>Survival: Kaplan-Meier<br>Costs: Mann-Whitney U test.<br>b) Yes (no difference found on clinical, OoL and pat sat baseline variables)<br>c) 3 months: n-int=76, n-control=74; 6 months: n-int=53, n-control= 58; 12 months: n-int=26, n-control=29<br>d) Not mentioned, but it was mentioned that no intervention group patients reverted to medical follow-up.	Int. group had less dyspnoea (p=0,03; a QoL score) and significantly higher satisfaction in each subscale at three months.<br><br>Int. group had longer time to symptomatic progression (p=0,01). Significant change in pattern of service use, but no difference in readmission rates. Significantly more patients in int. group died at home (p=0,04).<br>No difference in costs, and GP satisfaction.

? Not to be found in the article
***** Outcomes of interest: if primary and secondary was not indicated, "-" are used in front of each
[#]Validity account categorised as follows: ?: validity not mentioned at all; ref: reference(s) quoted; yes: it is mentioned that measure is validated
