# Supplementary material for: Case management used to optimize cancer care pathways: A systematic review
Source: BMC Health Serv Res. 2008 Nov 6;8:227. doi: 10.1186/1472-6963-8-227 (PMC2596122; doi:10.1186/1472-6963-8-227)
Supplement: Additional file 3 — Table 3: Important methodological aspects adapted from CONSORT [20,21]. [file 1472-6963-8-227-S3.pdf]

Table 3: *Important methodological aspects adapted from CONSORT [20,21]*

Ref	Methods paragraph<br>a) Sample size calculation? (yes/ no)<br>b) Period of recruitment listed? (yes/ no)<br>c) Described how patients were assessed for eligibility? (yes/ no)<br>d) Allocation concealment method (described/ not mentioned)<br>e) Blinding of those assessing outcomes (yes/ not mentioned)	Validity<br>a) Patient flow illustrated in diagram? (yes/ no)<br>b) Description of complete patient flow?<br>c) Non-participants mentioned (i.e. patients meeting criteria but refusing participation)? (yes/ no)<br>d) Non-participants characteristics compared with those included? (yes/ no)<br>If yes: potential for selection bias was discussed?<br>e) Accounting for participants not followed? (yes/ no)<br>If yes: Analyses performed to uncover potential skewed withdrawal? (yes/no)<br>Potential bias discussed?<br>f) Clearly described categorisation and follow-up of possible intervention-group patients not wanting the intervention? (yes/ no)
[19]	a) No<br>b) Yes (1/11/93-> 31/11/96)<br>c) Yes, both for patients and surgeons<br>d) Not mentioned<br>d) Yes	a) No diagram.<br>b) No, numbers of patients followed up by interview not available.<br>c) Yes (10 surgeons)<br>d) No<br>e) No<br>f) Yes (14, they were intention-to-treat analysed)
[23]	a) No<br>b) Yes (1995-97)<br>c) Yes<br>c) Not mentioned<br>d) Not mentioned	a) No<br>b) No, "Drop-out of intervention patients" and patients followed up with questionnaires not available.<br>c) Yes (85 did not wish to participate)<br>d) Participants were younger ($p<0.0001$) and were more likely to have invasive disease ($p=0.003$) than non-participants. Potential for selection bias not discussed.<br>e) No<br>f) No (and "intention-to-treat" not mentioned)
[25]	a) No<br>b) Yes (18-month period, dates not mentioned)<br>c) Yes<br>d) Not mentioned<br>e) Not mentioned	a) No<br>b) No. Numbers allocated to each group not stated and obscure flow of patients through each "arm".<br>c) No (more than 900 were asked to participate. Instead lung cancer registry cases in same County mentioned)<br>d) Yes, compared with above reg. "Diff. explainable" and were not considered a validity threat.<br>e) Yes, for the entire mass of included (111 withdrawn out of 166 randomized). Withdrawal reason for all 111 stated, but characteristics and group assignment of these not mentioned. No discussion.<br>f) No
[24]	a) No<br>b) Yes (February 1993- December 95)<br>c) No<br>d) Yes<br>e) Not mentioned	a) No<br>b) No, patients meeting criteria but not participating were not mentioned. Cause of attrition not accounted for.<br>c) No<br>d) No<br>e) No accounted for in numbers but causes for not responding qustionnaires not stated. No discussion.<br>f) No

Ref	Methods paragraph<br>a) Sample size calculation? (yes/ no)<br>b) Period of recruitment listed? (yes/ no)<br>c) Described how patients were assessed for eligibility? (yes/ no)<br>d) Allocation concealment method (described/ not mentioned)<br>e) Blinding of those assessing outcomes (yes/ not mentioned)	Validity<br>a) Patient flow illustrated in diagram? (yes/ no)<br>b) Description of complete patient flow?<br>c) Non-participants mentioned (i.e. patients meeting criteria but refusing participation)? (yes/ no)<br>d) Non-participants characteristics compared with those included? (yes/ no)<br>If yes: potential for selection bias was discussed?<br>e) Accounting for participants not followed? (yes/ no)<br>If yes: Analyses performed to uncover potential skewed withdrawal? (yes/no)<br>Potential bias discussed?<br>f) Clearly described categorisation and follow-up of possible intervention-group patients not wanting the intervention? (yes/ no)
[26]	a) No<br>b) No<br>c) No<br>d) Yes<br>e) Not mentioned	a) No<br>b) No, lack of explanation to numbers of participants not followed up.<br>c) No<br>d) No<br>e) No<br>f) Yes (cross-over both ways described in detail, intention to treat analysed)
[22]	a) Yes, estimate not presented.<br>b) No<br>c) Yes<br>d) Not mentioned<br>e) Not mentioned	a) No<br>b) No, unclear if 11 CM group patients who did not want CM were followed up or not (?)<br>c) Yes (125 patients)<br>d) No<br>e) Yes. No difference in attrition characteristics between intervention and control group patients. (Participants who had at least one follow-up were younger ($p<0.01$) and more likely to be female ($p<0.05$). Patients diagnosed from lymphoma, lung, pancreatic, or stomach cancer were less likely to be followed-up than breast cancer patients. Patients lost to follow-up were also more likely to have received palliative treatment.)<br>f) Yes, by number, but unclear if 11 case managed patients who refused CM services were followed up (they could be categorised under “attrition”?) and if followed up, in which group they were analysed.
[27]	a) Yes<br>b) Yes<br>c) No<br>d) Yes<br>e) Yes	a) Yes<br>b) Yes<br>c) Yes<br>d) No<br>e) Yes, but no analyses performed<br>f) Yes, it was mentioned that no intervention group patients reverted to medical follow-up.
